# DDHD1, but Not DDHD2, Suppresses Neurite Outgrowth in SH-SY5Y and PC12 Cells by Regulating Protein Transport From Recycling Endosomes

**DOI:** 10.3389/fcell.2020.00670

**Published:** 2020-07-23

**Authors:** Yuki Maemoto, Tomohiro Maruyama, Kazuaki Nemoto, Takashi Baba, Manae Motohashi, Akihiro Ito, Mitsuo Tagaya, Katsuko Tani

**Affiliations:** ^1^School of Life Sciences, Tokyo University of Pharmacy and Life Sciences, Hachioji, Japan; ^2^Department of Biological Informatics and Experimental Therapeutics, Graduate School of Medicine and Faculty of Medicine, Akita University, Akita, Japan

**Keywords:** endosome, phospholipase, neurite, phosphatidic acid, recycling

## Abstract

DDHD1 and DDHD2 are both intracellular phospholipases A_1_ and hydrolyze phosphatidic acid *in vitro*. Given that phosphatidic acid participates in neurite outgrowth, we examined whether DDHD1 and DDHD2 regulate neurite outgrowth. Depletion of DDHD1 from SH-SY5Y and PC12 cells caused elongation of neurites, whereas DDHD2 depletion prevented neurite elongation. Rescue experiments demonstrated that the enzymatic activity of DDHD1 is necessary for the prevention of neurite elongation. Depletion of DDHD1 caused enlargement of early endosomes and stimulated tubulation of recycling endosomes positive for phosphatidic acid-binding proteins syndapin2 and MICAL-L1. Knockout of DDHD1 enhanced transferrin recycling from recycling endosomes to the cell surface. Our results suggest that DDHD1 negatively controls the formation of a local phosphatidic acid-rich domain in recycling endosomes that serves as a membrane source for neurite outgrowth.

## Introduction

Phosphatidic acid (PA) forms signaling microdomains in cell membranes and is involved in a variety of physiological processes, including actin dynamics, membrane remodeling, apoptosis, and cell migration (Tanguy et al., 2019). Recent studies highlighted that PA also participates in invadopodia formation (Wang et al., 2017), podosome formation (Bolomini-Vittori et al., 2019), and the Hippo pathway (Han et al., 2018). PA is a key intermediate in glycerolipid biosynthesis and generated by enzymes such as phospholipase D (PLD) that hydrolyzes phospholipids, diacylglycerol (DAG) kinase that phosphorylates DAG, and lysoPA acyltransferase that acylates lysoPA. On the other hand, PA is metabolized to DAG by a PA phosphatases, Lipin 1. PA is converted to lysoPA by phospholipases A (PLA): PLA_1_ and PLA_2_ hydrolyze the sn-1 and sn-2 positions of PA, respectively.

The mammalian intracellular PLA_1_ (iPLA_1_) family (Tani et al., 2013) is composed of PA-preferring phospholipase A_1_ (PA-PLA_1_)/DDHD1 (Higgs et al., 1998), DDHD2/KIAA0725p (Nakajima et al., 2002), and p125/Sec23IP (Tani et al., 1999); the last of which, however, does not show significant phospholipase activity (Nakajima et al., 2002). Depending on their distribution, these enzymes could affect the structures and dynamics of distinct intracellular organelles (Tani et al., 2013). DDHD1 is the first identified iPLA_1_ and is highly expressed in the brain and testes (Higgs and Glomset, 1994). It can degrade not only PA but also phosphatidylinositol (PI) (Yamashita et al., 2010). Human genetic studies revealed that one of the causative mutations of hereditary spastic paraplegia (HSP) occurs in the DDHD1 gene (SPG28) (Tesson et al., 2012). HSP is an inherited neurodegenerative disorder characterized by length-dependent distal axonopathy, resulting in progressive lower limb spasticity and weakness (Blackstone, 2012). For SPG28, mitochondrial dysfunction has been suggested to be a crucial mechanism in its pathogenesis (Tesson et al., 2012; Liguori et al., 2014; Mignarri et al., 2016). Consistently, our previous study showed that DDHD1 regulates mitochondrial dynamics and that its ablation causes sperm malformation due to mitochondrial organization defects (Baba et al., 2014), although no SPG phenotype was seen in DDHD1 knockout (KO) mice (Baba et al., 2014). A lack of the HSP phenotype in DDHD1 KO mice was also reported very recently by Inloes et al. (2018). Moreover, they showed that DDHD1 ablation causes a decrease in lysoPI and a corresponding increase in PI in the brain, but not in the testes (Inloes et al., 2018).

Although DDHD1 is mostly cytosolic, DDHD2 is associated to some degree with membranes (Nakajima et al., 2002; Morikawa et al., 2009; Sato et al., 2010; Inoue et al., 2012) and shows affinity for PI4-phosphate (Inoue et al., 2012). Differing from DDHD1, DDHD2 is ubiquitously expressed (Nakajima et al., 2002). DDHD2 is also a causative gene for HSP (SPG54), and the patients exhibit a lipid peak in the brain detectable on magnetic resonance spectroscopy (Schuurs-Hoeijmakers et al., 2012; Doi et al., 2014). The HSP phenotype and neutral lipid accumulation of SPG54 could be reproduced in DDHD2 KO mice (Inloes et al., 2014; Maruyama et al., 2018), and DDHD2 was found to possess triacylglycerol lipase activity (Inloes et al., 2014) and DAG lipase activity (Araki et al., 2016; Aso et al., 2016). Although it has been suggested that neutral lipid accumulation in the brain is a cause of SPG54 (Inloes et al., 2014), our recent study involving DDHD2 KO mice and cells suggested that reactive oxygen species (ROS) production in mitochondria in motor neurons also likely contributes to cell apoptosis and the onset of SPG54 (Maruyama et al., 2018).

Phosphatidic acid and PA-producing enzyme PLD have been implicated in the regulation of neurite outgrowth (Hayakawa et al., 1999; Sung et al., 2001; Yoon et al., 2005; Zhang et al., 2005). Recycling endosomes, which are sorting station for recycling proteins such as transferrin receptor (TfR), play an important role in neurite outgrowth (Villarroel-Campos et al., 2014). Recycling endosomes exhibit tubular structures, which are characterized by the association of PA-binding proteins such as syndapin2 and MICAL-L1 (Giridharan et al., 2013) as well as Rab proteins (Kobayashi and Fukuda, 2013; Kobayashi et al., 2014; Etoh and Fukuda, 2019). We were therefore interested in whether DDHD1 and DDHD2, both of which exhibit PLA_1_ activity toward PA *in vitro* and in cells when ectopically expressed (Higgs and Glomset, 1994; Higgs et al., 1998; Nakajima et al., 2002; Yamashita et al., 2010; Inoue et al., 2012; Inloes et al., 2018), regulate neurite elongation. In the present study, we showed that DDHD1, but not DDHD2, negatively regulates neurite outgrowth. Depletion of DDHD1 caused an endosomal defect with abnormal protein recruitment to tubular recycling endosomes.

## Materials and Methods

### Plasmid Construction and Virus Production

The pMRX-IRES-puro and pMRX-IRES−bsr vectors (Saitoh et al., 2003) were kindly provided by S. Yamaoka (Tokyo Medical and Dental University, Tokyo, Japan). The pMRX-IRES-puro-DEST-mCherry and pMRX-IRES-bsr-DEST-EGFP vectors (Imai et al., 2016) were generously provided by T. Yoshimori (Osaka University). mRFP-PASS and mRFP-PASS4E (Zhang et al., 2014) were generously provided by M. Frohman (Stony Brook University). For retroviral expression plasmids, cDNA coding siRNA-resistant DDHD1 (Baba et al., 2014) was amplified by PCR and inserted into the *BamH*I and *EcoR*I sites of pENTR1A using an In-Fusion^®^ Advantage PCR Cloning Kit. DNA fragments of DDHD1 and DDHD1^S537A^ in pENTR1A plasmids were transferred to the pMRX-IRES-puro-DEST-mCherry or pMRX-IRES-bsr-DEST-EGFP vector using an LR reaction, respectively. Expression plasmids for DDHD2-FLAG and DDHD2^S351A^-FLAG were described previously. Recombinant retroviruses were prepared as previously described (Maemoto et al., 2014). Plat-E cells were generously provided by T. Kitamura (Tokyo University) (Morita et al., 2000).

### RNA Interference Experiment

siRNAs were purchased from Japan Bio Services. The siRNA targeting sequences used for SH-SY5Y or HeLa cells were as follows: Luciferase siRNA, CGTACGCGGAATACTTCGA; DDHD1 siRNA#2, AAGCCACATTAGAAGACAAGC; DDHD1 siRNA#5, AAGAGTTGCCTGATGAACGAT. DDHD2 siRNA#2, AAGAAAGAAGAUAUUAAACUA; and DDHD2 siRNA#3, AA GGAGAAAGUAGAUAAGGAA. The siRNA targeting sequences used for PC12 cells were as follows: Luciferase siRNA, CGTAC GCGGAATACTTCGA; and DDHD1 GGAGGAATGTTGTTC TCAA. Cells were transfected with siRNA at a final concentration of 100 nM using Lipofectamine RNAiMAX (Invitrogen) according to the manufacturer’s protocol, and cells were fixed and processed at 72 h after transfection.

### Cell Culture and Retroviral Infection

SH-SY5Y and HeLa cells were cultured in Dulbecco’s modified Eagle’s medium (DMEM) supplemented with 10% fetal bovine serum (FBS) and penicillin/streptomycin. PC12 cells were maintained in Dulbecco’s modified Eagle’s medium (DMEM) supplemented with 10% FBS and 10% horse serum and penicillin/streptomycin. All cells were cultured at 37°C under humidified air containing 5% CO_2_. For retroviral infection, cells were treated with a virus solution for 24 h and then washed with medium.

### Establishment of DDHD1 KO Cells

A pair of gRNA oligonucleotides for each targeting site was annealed and ligated to the *Bbs*I-treated pSpCas9(BB)-2A-GFP (pX458) vector (Ran et al., 2013), and the sequences of the gRNAs were verified by sequencing analysis. To generate KO cell lines, the CRISPR design tool^1^ was used to identify gRNA sequences and the following sequence was used as the target gRNA: 5′- TGAGTCGAAACCATGTGGAC -3′. HeLa cells were transfected with the pX458 vector and one day after transfection, cells that highly expressed GFP with Cas9 were isolated by FACS (SH800, Sony) and cultured. The edited locus was amplified using primers (5′- CCCTATCCATTACTTGCTTCAC -3′ and 5′- CCTTGATGAACACATGTCAACTC -3′) and confirmed by Sanger sequencing.

### Antibodies and Reagents

Monoclonal antibodies against DDHD1 (12D10) were raised previously (Baba et al., 2014). Polyclonal antibodies against syndapin2 and α-tubulin were obtained from Abgent and Sigma-Aldrich, respectively. Polyclonal antibodies against MICAL-L1 and Rab13 were purchased from Abcam. Monoclonal antibodies against MICAL-L1, EEA1, and TfR were obtained from Abnova, BD Transduction Laboratories, and Sigma-Aldrich, respectively. Alexa Fluor 488-conjugated goat anti-mouse antibodies and Alexa Fluor 594 goat anti-mouse antibodies were purchased from Invitrogen. The following reagents were used: NGF (Alomone Labs); RA, unlabeled-Tfn, deferoxamine, R59949, FITC-phalloidin and TRITC-phalloidin (Sigma-Aldrich); CAY1059 and CAY10594 (Cayman Chemical); Alexa488-Tfn and TRITC-Tfn (Thermo Fisher Scientific); and EGF (Invitrogen).

### Immunofluorescence Analyses

Immunofluorescence images were analyzed as described previously (Inoue et al., 2012; Baba et al., 2013). An Olympus FluoView 1000 or FluoView 1200 laser scanning microscope was used for confocal microscopy. For immunostaining, cells were fixed with 4% paraformaldehyde for 20 min and then permeabilized with 50 ng/ml digitonin for 10 min. After 30 min blocking with 2% bovine serum albumin/PBS for 30 min, the cells were subjected to primary antibodies for 1 h followed by secondary antibodies for 1 h. After washing with PBS, the cells were mounted on a slide glass with a mounting solution (100 mM Tris–HCl, pH 8.5, 25% (w/v) glycerol, 10% (w/v) Mowiol 4-48).

### Stimuli for Neurite Outgrowth

For SH-SY5Y cells, cells were incubated with 10 μM RA in DMEM supplemented with 2% FBS for 72 h at 37°C after each treatment. At 72 h after siRNA transfection, PC12 cells were stimulated with 100 nM NGF in DMEM supplemented with 1% FBS and 0.1 mM L-glutamine.

### Analysis of Neurite Length

Signals for FITC-phalloidin, TRITC-phalloidin or mCherry were obtained by confocal microscopy visualized and analyzed with ImageJ software. The average length of longest neurite in each cell was determined between experiments. The results are expressed as means and standard deviation (SD) for at least three independent experiments. Statistically significant differences were determined using Tukey multiple comparison tests or Student’s *t* test as appropriate. Differences were considered significant if *P* < 0.05.

### Tfn Recycling Assay

Cells were split and starved with serum-free DMEM for 2 h and then incubated with 5 μg/ml Alexa488-Tfn for 1 h. Uptake was stopped with acid wash buffer (50 mM MES and 150 mM NaCl, pH 5.5) to remove cell surface Tfn, and the cells were then incubated with DMEM containing 100 μg/ml label-free Tfn and 100 μM deferoxamine for the indicated times. To stop the recycling, the cells were chilled on ice and washed with ice-cold acid wash buffer. The cells were then fixed with 4% paraformaldehyde in PBS for 20 min.

### EGF Receptor-Degradation Assay

The EGF degradation analysis was described previously (Maemoto et al., 2014), Briefly, one day after HeLa cells had been seeded, the cells were serum-starved for 3 h and then stimulated with 100 ng/ml EGF at 37°C for the indicated times. The cells were harvested with Laemmli sample buffer. The intensity of immunoreactive signals was quantified with ImageJ.

## Results

### DDHD1 Negatively Regulates Neurite Outgrowth

**FIGURE 1:**
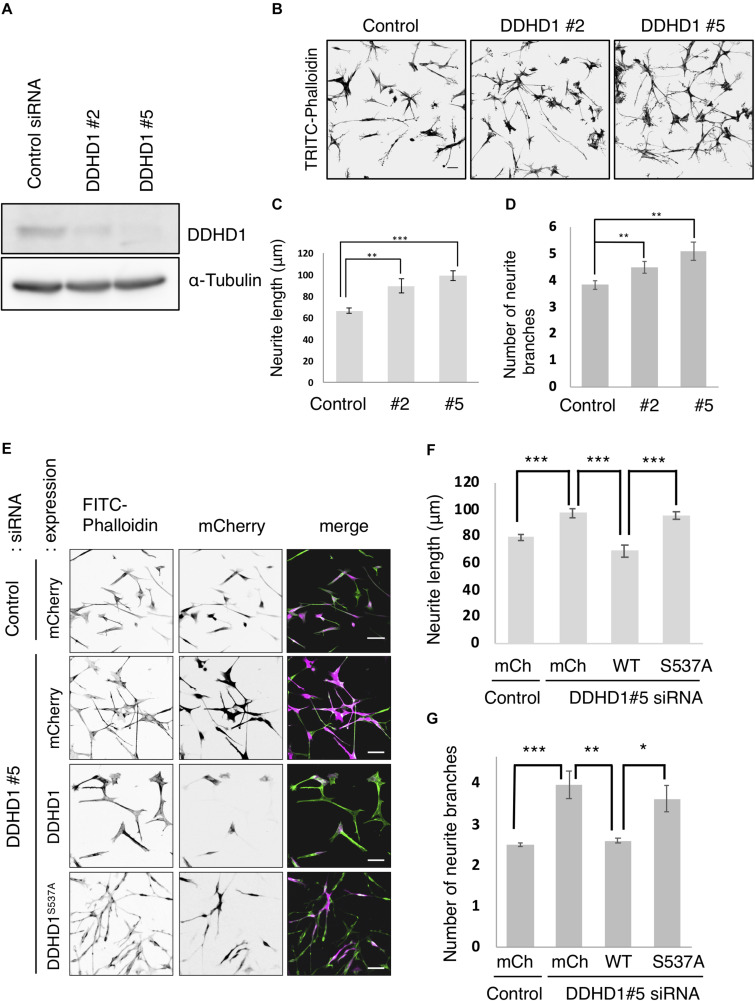
Knockdown of DDHD1 induces enhanced neurite outgrowth in SH-SY5Y cells. **(A)** SH-SY5Y cells treated with luciferase siRNA (control siRNA), DDHD1 siRNA#2, or DDHD1 siRNA#5 were analyzed by Western blotting with the indicated antibodies. **(B)** At 72 h after siRNA transfection, SH-SY5Y cells were subjected to RA treatment for 72 h to induce neurite outgrowth. The cells were fixed and stained with TRITC-phalloidin. Scale bars, 50 μm. **(C,D)** Quantification of the data in **(B)**. The graph shows the average length of the longest neurite tubule **(C)** and the number of neurite branches **(D)** in each cell. **(E)** SH-SY5Y cells were treated with DDHD1 siRNA#5 or control siRNA for 48 h, and then infected with the indicated retroviruses. At 24 h after infection, the cells were treated with RA for 72 h. The fluorescent signals for FITC-phalloidin, mCherry and merged image are shown. Scale bars, 50 μm. **(F,G)** Quantification of the data in **(E)**. The average length of the longest neurite tubule **(F)** and the number of neurite branches **(G)** in each cell was measured and is shown in the graph. At least 50 cells were measured in each experiment. Statistic values are expressed as means for three independent experiments ± S.D. *p < 0.05; **p < 0.01; ***p < 0.001 (Tukey test).

Previous studies revealed that PA on the recycling endosomes forms a microdomain (Giridharan et al., 2013; Bahl et al., 2016; Henmi et al., 2016) and that a PA-binding protein is necessary for neurite outgrowth (Kobayashi and Fukuda, 2013; Kobayashi et al., 2014). Since DDHD1 is highly expressed in neuronal cells, we examined the effect of DDHD1 depletion on neurite outgrowth in neuronal cells. At 72 h after siRNA treatment of human neuroblastoma SH-SY5Y cells, neurite outgrowth was induced with RA. Western blot analysis verified substantial reductions in the level of DDHD1 by two different siRNAs (DDHD1#2 and #5) (Figure 1A). Upon DDHD1 depletion, neurite tubules appeared to be elongated and branched (Figure 1B). We determined the length of the longest neurite extending from a cell, and found that the enhancement of neurite outgrowth and of the number of branches by DDHD1 depletion was statistically significant (Figures 1C,D). To exclude the possibility of off-target effects and to determine whether the phospholipase activity of DDHD1 plays a role in neurite outgrowth, siRNA-resistant mCherry-DDHD1 wild-type and enzymatic inactive mCherry-DDHD1^S537A^, in which catalytic residue Ser537 was replaced by Ala, were expressed by infection with recombinant viruses encoding the proteins (Baba et al., 2014). Judging from the mCherry fluorescence intensity, the expression level of mCherry-DDHD1 wild-type may be lower than that of the mutant. Nevertheless, neurite lengthening and branching was suppressed by the wild-type protein, but not the mutant protein (Figures 1E–G). DDHD1 depletion and rescue experiments involving rat pheochromocytoma PC12 cells which had been stimulated with NGF gave similar results (Supplementary Figure S1). These results suggest that the enzymatic activity toward PA could negatively regulate neurite outgrowth. We performed neurite elongation assays using DDHD2-depleted cells. Contrary to that of DDHD1, depletion of DDHD2 suppressed neurite outgrowth (Supplementary Figures S2A,B), which was reversed by expression of wild-type DDHD2, but not enzymatically inactive DDHD2^S351A^ (Supplementary Figures S2C,D). In addition, this suppression was reversed by *N*-acetylcysteine, a ROS scavenger (Supplementary Figures S2E,F), which is consistent with our previous finding that depletion of DDHD2 promotes ROS production and cell apoptosis (Maruyama et al., 2018).

Given that DDHD1, when ectopically expressed, exhibits PLA_1_ activity toward PI as well as PA *in vitro* and in cells (Yamashita et al., 2010; Inoue et al., 2012), the DDHD1 depletion effect on neurite outgrowth may not be attributable to PA turnover. To determine whether or not the amount of PA affects neurite outgrowth, SH-SY5Y cells were treated with a DAG kinase inhibitor (R59949) and PLD inhibitors (CAY10593 and 10594), both of which are supposed to inhibit PA production (Supplementary Figure S3A). The average neurite length of control cells was significantly greater than that of cells treated with a DAG kinase inhibitor or PLD inhibitors (Supplementary Figures S3B,C), consistent with our notion that PA upregulation by DDHD1 depletion enhances the neurite outgrowth.

### DDHD1 Knockdown Affects Endosomal Structure

**FIGURE 2:**
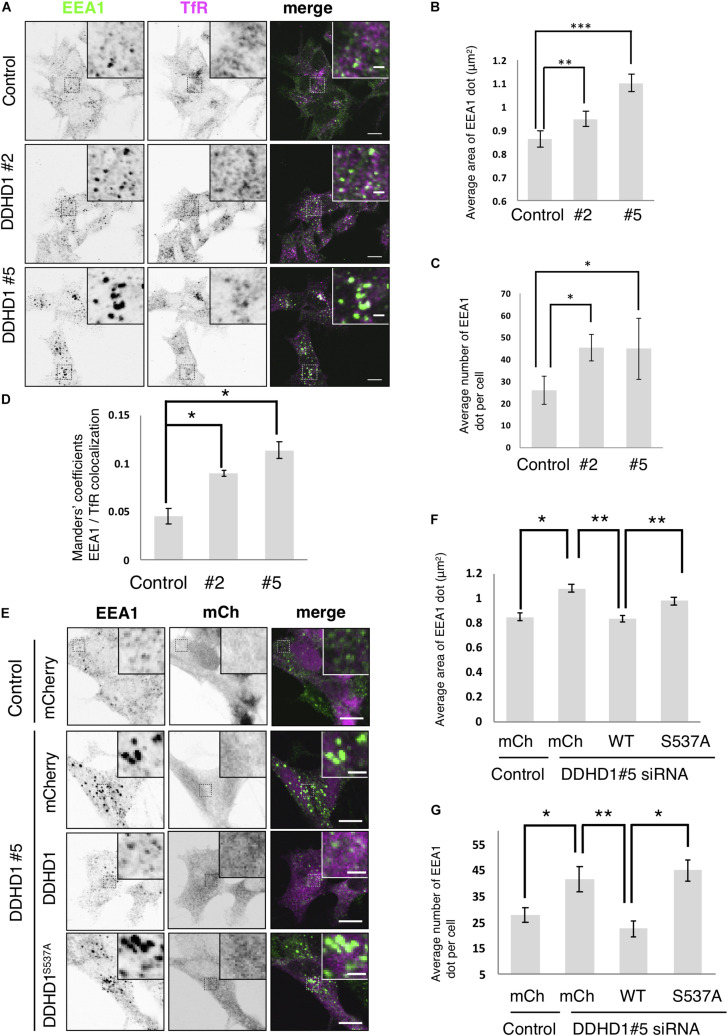
Effects of DDHD1 knockdown on endosomal morphology in SH-SY5Y cells. **(A)** SH-SY5Y cells were treated with the indicated siRNAs for 72 h, and then stained with antibodies against EEA1 and TfR. Higher magnification views of the boxed areas are shown in the inset. Scale bars, 10 μm; inset, 2 μm. **(B,C)** Quantification of the data in panel **(A)**. The average area **(B)** and number **(C)** of early endosomes in each cell were measured under each condition. **(D)** Manders’ colocalization analysis of the data in panel **(A)**. **(E)** SH-SY5Y cells were treated with DDHD1 siRNA#5 or control siRNA for 48 h, and then infected with the indicated retroviruses. At 24 h after infection, the cells were fixed and stained with an antibody against EEA1. Scale bars, 10 μm; inset, 2 μm. **(F,G)** Quantification of the data in panel **(E)**. The average area **(F)** and number **(G)** of early endosomes in each cell were calculated. At least 30 cells were examined in each experiment. Values are expressed as means for three independent experiments ± S.D. *p < 0.05; **p < 0.01; ***p < 0.001 (Tukey test).

To explore the function of DDHD1 in endosomes in neuronal cells, we first observed the morphology of early endosomes (visualized with an early endosomal marker, EEA1) in DDHD1-depleted SH-SY5Y cells. On immunofluorescence microscopy, enlarged early endosomes with an increase in their number were detected in DDHD1-depleted cells compared to in control wild-type cells (Figure 2A). The quantitative analysis confirmed this notion (Figures 2B,C). Manders’ colocalization analysis revealed enhanced colocalization of EEA1 and a recycling/early endosome marker, TfR (Figure 2D), suggesting the merging of early and recycling endosomes in DDHD1-depleted cells. This idea was supported by the finding that Rab13, a marker for recycling endosomes/*trans*-Golgi network (Nokes et al., 2008), is colocalized with EEA1 in DDHD1-depleted cells (Supplementary Figure S4), disfavoring the possibility that cargo (TfR) is selectively retained in early endosomes as a result of DDHD1 depletion.

Next, we performed knockdown rescue experiments. At 48 h after siRNA treatment, cells were infected with retrovirus for the expression of mCherry, mCherry-DDHD1 wild-type, and mCherry-DDHD1^S537A^. Expression of mCherry-DDHD1 wild-type reversed the enlargement and increased average number of early endosomes caused by siRNA, but the PLA_1_-inactive mutant or mCherry could not (Figures 2E–G). As a control, we depleted DDHD2 and found that DDHD2 depletion did not affect the endosomal structure (Supplementary Figure S5), consistent with the finding that DDHD2 depletion did not induce neurite growth, but rather inhibited it (Supplementary Figure S2).

### Depletion of DDHD1 Enhances the Localization of PA-Binding Proteins on Recycling Endosomes

**FIGURE 3:**
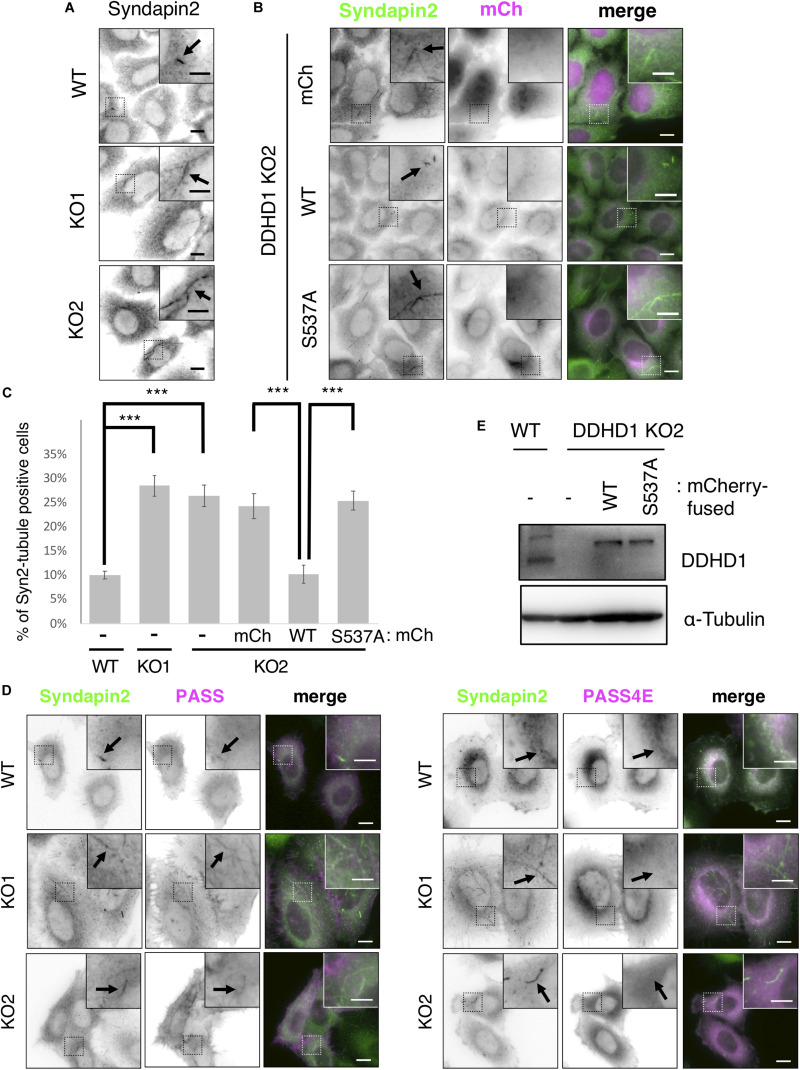
Effect of DDHD1 KO on syndapin2-positive recycling tubules in HeLa cells. **(A,B)** HeLa cells and DDHD1 KO HeLa cells **(A)**, and DDHD1 KO HeLa cells-2 stably expressing mCherry, mCherry-DDHD1, or mCherry-DDHD1^S537A^ **(B)** were fixed and stained with anti-syndapin2 antibody. Higher magnification views of the boxed areas are shown in the inset. Scale bars, 10 μm; inset, 5 μm. **(C)** Quantification of the data in panels **(A,B)**. The percentages of cells containing syndapin2-positive tubules of over 3 μm are shown in the graph. At least 50 cells were examined in each experiment. ***p < 0.01 (Tukey test). **(D)** Expression level of endogenous DDHD1 in HeLa cells, mCherry-DDHD1, and mCherry-DDHD1^S537A^ in DDHD1 KO HeLa cells were analyzed by Western blotting with the indicated antibodies. **(E)** HeLa cells and DDHD1 KO HeLa cells were transfected with plasmid for mRFP-PASS or mRFP-PASS 4E. At 24 h after transfection, cells were stained with anti-syndapin2 antibody. Higher magnification views of the boxed areas are shown in the inset. Scale bars, 10 μm; inset, 5 μm.

To gain an insight into the mechanism of neurite outgrowth, we focused on a PA-binding-proteins, MICAL-L1, which is involved in neurite outgrowth through its association with a PA-rich domain on recycling endosomes (Kobayashi et al., 2014), and syndapin2. After NGF stimulation of PC12 cells, Rab35, MICAL-L1, Rab8, and ACAP2 are sequentially recruited to recycling endosomes, and this process is important for neurite outgrowth (Kobayashi and Fukuda, 2013). The recruitment of MICAL-L1 and syndapin2 to PA-enriched endosomes is important for tubular recycling endosome biogenesis (Giridharan et al., 2013). We first examined the localization of syndapin2 in DDHD1 KO HeLa cells. DDHD1 KO was confirmed by DNA sequencing (Supplementary Figures S6A,B) and Western blotting (Supplementary Figure S6C). Enlarged early endosomes were observed in DDHD1 KO HeLa cells (Supplementary Figure S6D), like those observed in DDHD1 knockdown SH-SY5Y cells (Figure 2). Immunofluorescent analysis of syndapin2 revealed that the percentage of cells that possess syndapin2-positive tubular recycling endosomes was increased in DDHD1 KO HeLa cells compared to in control cells (Figures 3A,C). These increased syndapin2-positive structures were found to be colocalized with a PA-sensor, mRFP-PASS, but not mRFP-PASS 4E, a mutant version of PASS (Zhang et al., 2014) that does not bind PA (Figure 3D). This result suggests that DDHD1 ablation causes a local increase in PA-level on endosomes, which in turn promotes the formation of syndapin2-positive structures. Expression of wild-type DDHD1 suppressed tubulation of syndapin2-positive recycling endosomes, whereas that of DDHD1^S537A^ did not (Figures 3B,C), corroborating that the phospholipase activity of DDHD1 is responsible for the suppression of syndapin2-tubulation. Similar expression levels of mCherry-DDHD1 wild-type, DDHD1^S537A^, and endogenous DDHD1 were confirmed by Western blotting (Figure 3E).

Endosome enlargement (Supplementary Figure S7B) and MICAL-L1-positive tubule formation (Supplementary Figures S7C,D) were also observed in HeLa cells whose DDHD1 was knocked down using the same siRNA as that used for SH-SY5Y (Supplementary Figure S7A). Expression of mCherry-DDHD1 wild-type, but not mCherry-DDHD1^S537A^, suppressed tubulation of MICAL-L1-positive tubular structures (Supplementary Figures S7E,F).

### Depletion of DDHD1 Enhances the Recycling of Tfn to the Cell Surface

**FIGURE 4:**
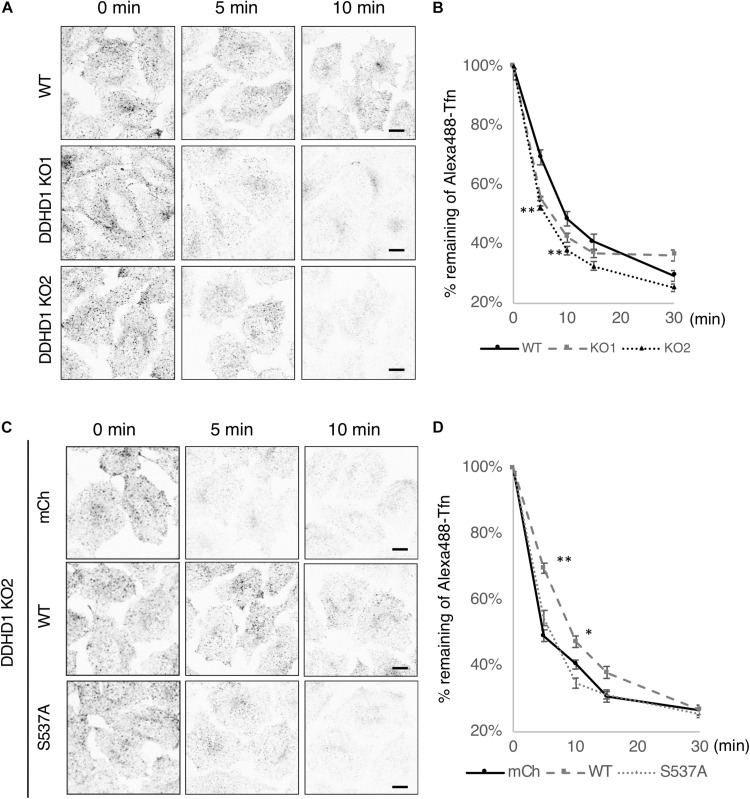
Effects of DDHD1 activity on Tfn recycling in HeLa cells. **(A,C)** Wild-type or DDHD1 KO HeLa cells **(A)** or DDHD1 KO HeLa cells expressing mCherry, mCherry-DDHD1, or mCherry-DDHD1^S537A^ **(C)** were subjected to Alexa488-Tfn recycling assay for the indicated times as described under Experimental Procedures. Scale bars, 10 μm. **(B,D)** the percentages of remaining Alexa488-Tfn signals compared to the fluorescent signals at 0 min are shown in the graph. Values are expressed as means for three independent experiments ± S.D. *p < 0.05; **p < 0.01; (Tukey test).

It has been reported that depletion of PA inhibits Tfn recycling to the cell surface (Giridharan et al., 2013). If PA accumulates on recycling endosomes and thereby stabilizes their tubular structure upon DDHD1 ablation, the rate of Tfn recycling could be affected. To examine this possibility, Tfn recycling assay was performed using DDHD1 KO HeLa cells. The fluorescent intensities of Alexa488-Tfn of DDHD1 KO cells were lower than those of control wild-type cells at 5, 10, and 15 min after the initiation of recycling (Figures 4A,B). To understand the importance of the enzymatic activity of DDHD1 for recycling, we performed recycling assay using DDHD1 KO HeLa cells expressing mCherry, mCherry-DDHD1 wild-type, or mCherry-DDHD1^S537A^. The enhanced rate of Tfn release by DDHD1 KO HeLa cells was suppressed by the expression of mCherry-DDHD1 wild-type at any time investigated, whereas no such effect was seen for mCherry-DDHD1^S537A^ (Figures 4C,D). No significant effect on TfR recycling was observed when DDHD2 was knocked out (Supplementary Figure S8). The effect of DDHD1 depletion appeared to be only on the recycling process because the rate of EGF receptor downregulation was indistinguishable between control wild-type HeLa cells and DDHD1 KO cells (Supplementary Figure S9).

### Depletion of DDHD1 Enhances MICAL-L1 Recruitment to Recycling Endosomes in PC12 Cells

**FIGURE 5:**
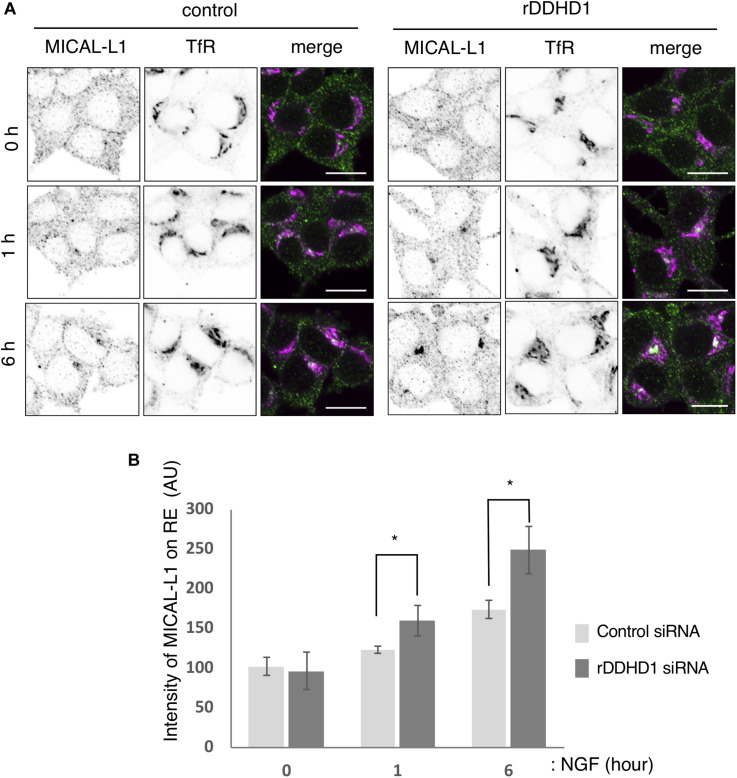
Enhancement of MICAL-L1 recruitment to the recycling endosomes by rDDHD1 knockdown in PC12 cells. **(A)** At 72 h after siRNA treatment, PC12 cells were stimulated with NGF for the indicated times. The cells were fixed and stained with antibodies against TfR and MICAL-L1. Scale bars, 10 μm. **(B)** MICAL-L1 signals on recycling/early endosomes (RE) visualized with anti-TfR antibodies were calculated for each period and are shown in the graph. At least 100 cells were examined in each experiment. Values are expressed as means for four independent experiments ± S.D *p < 0.05 (Student’s *t* test).

As reported, Rab35 and MICAL-L1 were recruited to recycling endosomes on NGF treatment in PC12 cells. At 1 and 6 h after NGF stimulation, the intensity of the MICAL-L1 fluorescent signal on TfR positive-recycling endosomes was higher in DDHD1 knockdown cells than that in control cells (Figures 5A,B). These results imply that knockdown of DDHD1 accelerates the recruitment of MICAL-L1 and perhaps membrane supply to the neurite ends.

## Discussion

Phosphatidic acid with a cone-shaped geometry is thought to induce membrane curvature and thereby participate in membrane remodeling (Mukherjee and Maxfield, 2000; McMahon and Gallop, 2005; Antonescu et al., 2010). PA also forms microdomains in organelles including endosomes and recruits certain proteins (Tanguy et al., 2019). In neuronal cells, PA has been reported to be involved in neurite outgrowth by recruiting PA-binding proteins such as MICAL-L1, followed by EHD1 recruitment, to endosomes (Kobayashi and Fukuda, 2013), where EHD1 likely carries out scission to facilitate vesicle transport and recycling to the plasma membrane (Giridharan et al., 2013). Although the role of PA-forming enzymes such as PLD in neurite outgrowth has been extensively studied (Hayakawa et al., 1999; Kanaho et al., 2009), the function of PA-degrading enzymes in this process remains unknown. In this study, we demonstrated that DDHD1, an HSP-related protein that exhibits PLA_1_ activity toward PA (Higgs and Glomset, 1994; Higgs et al., 1998; Yamashita et al., 2010; Inloes et al., 2018) and is highly expressed in the brain (Higgs et al., 1998; Baba et al., 2014), regulates neurite outgrowth.

Depletion of DDHD1 enhanced neurite outgrowth in SH-SY5Y cells (Figure 1) and PC12 cells (Supplementary Figure S1). Rescue experiments showed that the enzymatic activity of DDHD1 is responsible for the suppression of neurite outgrowth. DDHD1-depleted cells exhibited enlarged endosomes (Figure 2 and Supplementary Figure S6D) with elongated syndapin2- and MICAL-L1-positive structures (Figure 3 and Supplementary Figure S7). Tfn recycling assay demonstrated that the rate of recycling to the plasma membrane was significantly increased in DDHD1-depleted HeLa and MEF cells (Figure 4 and Supplementary Figure S8). The correlation between enhanced neurite outgrowth and endosomal recycling is consistent with the idea that the tubulovesicular recycling endosomes travel toward the cell periphery in neurites to act as a membrane source (Prekeris et al., 1999).

Based on lipidomic analysis of DDHD1 knockout mice, Inloes et al. suggested that DDHD1 is a primary PI lipase in the brain; no significant change in the PA level was observed in knockout mice (Inloes et al., 2018). In our previous study, however, we demonstrated using a PA sensor that DDHD1 ablation induced an increase in the PA level in a microdomain on the mitochondrial surface, although the total amount of PA did not change (Baba et al., 2014). In the present study we demonstrated that enlarged recycling endosomes are enriched in PA, as revealed by another PA sensor (Figure 3D), suggesting that an increase in the PA level in a microdomain on recycling endosomes. Our previous results combined with the present observation that inhibition of PLD and DAG kinase by specific inhibitors suppressed neurite outgrowth (Supplementary Figure S3) may suggest that the activity of DDHD1 toward PA is responsible for neurite outgrowth suppression. However, the increase in the PA level concomitant with DDHD1 ablation in the mouse brain might be under the detection limit of mass spectrometric analysis. Unfortunately, as PI sensors are not available, we cannot exclude the possibility that DDHD1 may regulate neurite outgrowth by metabolizing PI in a microdomain on recycling endosomes.

DDHD1 is principally localized to the cytosol (Higgs et al., 1998; Yamashita et al., 2010; Baba et al., 2014) and, unlike other PLA_1_ family members, has no ability to bind to phospholipids including PA (Inoue et al., 2012). At present it is not clear how DDHD1 associates with endosomes and performs its action. One possibility is that it gets into contact with endosomes through diffusion and then immediately dissociates after completion of the enzymatic action. Alternatively, endosomal protein(s) may assist the binding of DDHD1 to endosomes. Even in the latter case, their affinity for DDHD1 may be very low, given the predominantly cytosolic localization of DDHD1.

Recent studies have revealed that mutations in the PA-PLA_1_/DDHD1 gene cause HSP (Tesson et al., 2012) and amyotrophic lateral sclerosis (Wu and Fan, 2016). Lipin 1 metabolizes PA, and its mutations in mice (Nadra et al., 2008; Douglas et al., 2009) and rats (Mul et al., 2011) induce hind limb paralysis, a typical symptom of HSP. However, no SPG phenotype was seen in DDHD1 KO mice (Baba et al., 2014). This may be consistent with our present observation that DDHD1 depletion in cultured neuronal cells neither caused cell death nor inhibited neurite outgrowth, but rather enhanced neurite outgrowth. The present observation might predict the elongation of axons in DDHD1 knockout mice, but this was found not to be the case (data not shown). This is likely due to the fact that *in vivo* axonal elongation is regulated by many other factors other than the PA level in recycling endosomes such as axon elongation stimulators and inhibitors. However, as DDHD1 expression was found to be suppressed in embryos and expressed on postnatal day 8 (data not shown), the time when striking development of the brain’s fiber tracts as well as remodeling of cortical and subcortical structure occurs, DDHD1 may have some functions in brain development, especially in humans in which its mutation causes HSP (Tesson et al., 2012).

In contrast to DDHD1, DDHD2 KO mice exhibited a typical HSP phenotype, including age-dependent apoptosis of motor neurons in the spinal cord (Maruyama et al., 2018). DDHD2 depletion was found to not inhibit neurite outgrowth or enhance the recycling pathway (Supplementary Figures S2, S8). Therefore, although DDHD1 and DDHD2 exhibit PLA_1_ activity toward PA *in vitro* (Higgs and Glomset, 1994; Higgs et al., 1998; Yamashita et al., 2010; Inloes et al., 2018), these physiological roles are different.

## Conclusion

In conclusion, our data point to a novel role for DDHD1 as a suppressor of neurite outgrowth in cultured neuronal cells. DDHD1 regulates neurite outgrowth by regulating the PA level in a microdomain on recycling endosomes.

## Data Availability Statement

The raw data supporting the conclusions of this article will be made available by the authors, without undue reservation.

## Author Contributions

YM designed the study and conducted the experiments. YM and MT wrote the manuscript. KN, YM, TM, and MM performed the experiments. All authors analyzed the results and approved the final version of manuscript. KT and AI provided supervision.

## Conflict of Interest

The authors declare that the research was conducted in the absence of any commercial or financial relationships that could be construed as a potential conflict of interest.

## Footnotes

1 http://crispr.mit.edu

## Abbreviations

DAG: diacylglycerol
DMEM: Dulbecco’s modified Eagle’s medium
EGF: epidermal growth factor
FBS: fetal bovine serum
HSP: hereditary spastic paraplegia
iPLA_1_: intracellular PLA_1_
KO: knockout
lysoPA: lysophosphatidic acid
lysoPI: lysophosphatidylinositol
MEF: mouse embryonic fibroblast
NGF: nerve growth factor
PA: phosphatidic acid
PI: phosphatidylinositol
PLA: phospholipase A
PLD: phospholipase D
RA: retinoic acid
ROS: reactive oxygen species
SPG: spastic paraplegia
Tf: transferrin
TfR: Tf receptor.
