# Supplementary material for: DDHD1, but Not DDHD2, Suppresses Neurite Outgrowth in SH-SY5Y and PC12 Cells by Regulating Protein Transport From Recycling Endosomes
Source: Front Cell Dev Biol. 2020 Jul 23;8:670. doi: 10.3389/fcell.2020.00670 (PMC7396612; doi:10.3389/fcell.2020.00670)
Supplement: Supplementary file 1 [file Data_Sheet_1.PDF]

Supplementary Material

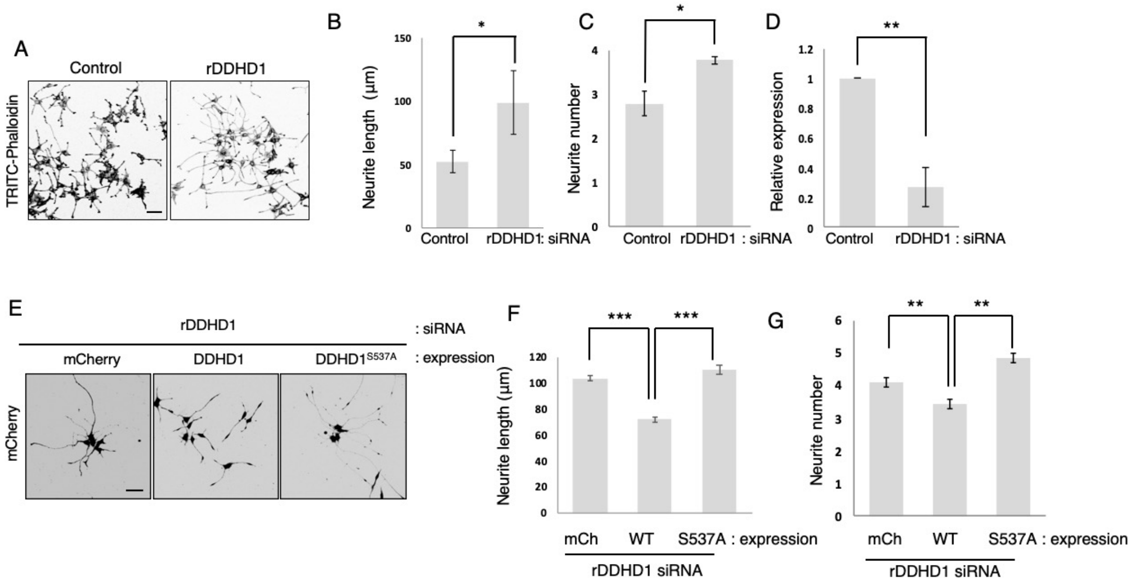

Supplementary Figure 1. Knockdown of rDDHD1 induces enhanced neurite outgrowth in PC12 cells. (A) At 72h after siRNA treatment, PC12 cells were subjected to NGF treatment for 72 h to induce neurite outgrowth. The cells were fixed and stained with TRITC-phalloidin. Scale bars, 50 μm. **(B,C)** Quantification of the data in (A). The graphs show the average length of the longest neurite tubule (B) and number (C) of neurite in each cell. *, $p < 0.05$ (Student's t test). **(D)** The expression level of rDDHD1 mRNA was measured by realtime PCR. **, $p < 0.01$ (Student's t test) **(E)** PC12 cells were treated with rDDHD1 siRNA for 48 h, and then infected with the indicated retroviruses. At 24 h after infection, the cells were treated with NGF for 72 h. The fluorescent signals for mCherry are shown. Scale bars, 50 μm. **(F,G)** Quantification of the data in (E). The graphs show the average length of the longest neurite tubule (F) and number (G) of neurite tubules. At least 50 cells were examined in each experiment. **, $p < 0.01$ (Tukey test). Values are expressed as means for three independent experiments ± S.D.

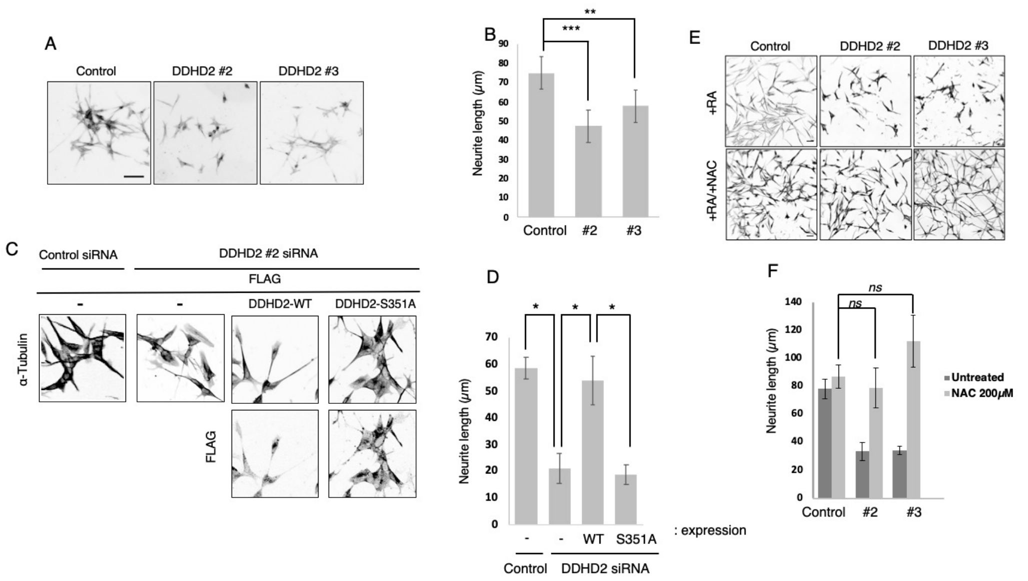

Supplementary Figure 2. Knockdown of DDHD2 inhibits neurite outgrowth via ROS production in SH-SY5Y. (A) SH-SY5Y cells treated with luciferase siRNA (control siRNA), DDHD2 siRNA#2, or DDHD2 siRNA#3 were subjected to RA treatment for 72 h to induce neurite outgrowth. The cells were fixed and stained with TRITC-phalloidin. Scale bars, 50 µm. **(B)** Quantification of the data in (A). The graph shows the average length of the longest neurite tubules. **(C)** SH-SY5Y cells were treated with DDHD2 siRNA#2 and control siRNA for 48 h, and then infected with the indicated retroviruses. At 24 h after infection, the cells were treated with RA for 72 h and then stained with α-tubulin and FLAG antibodies. Scale bars, 50 µm. **(D)** Quantification of the data in (C). The average length of the longest neurite tubules was measured and is shown in the graph. **(E)** At 48 h after DDHD2 siRNA transfection, SH-SY5Y cells were treated with RA for 72 h with or without 200 µM N-acetylcysteine (NAC). Scale bars, 50 µm. **(F)** Quantification of the data in (E). The average length of the longest neurite tubules was measured and is shown in the graph. At least 50 cells were examined in each experiment. Values are expressed as means for three independent experiments ± S.D. *, p <0.05; **, p <0.01; ***; p <0.001, ns; not significant (Tukey test).

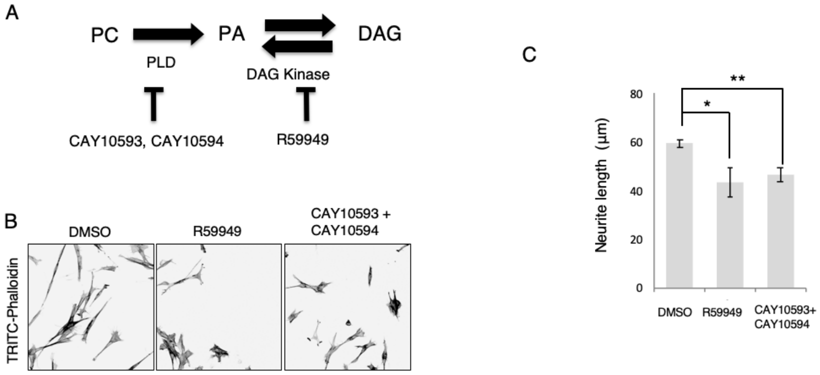

Supplementary Figure 3. Effects of depletion of PA on neurite outgrowth in SH-SY5Y cells. (A) The steps of inhibition by inhibitors in the metabolic pathway of PA synthesis. **(B)** SH-SY5Y cells were treated with DMSO, R59949 (0.25 µM), or CAY10593 and CAY10594 (each 2.5 µM) with RA in DMEM containing 2% FBS for 72 h. The cells were stained with TRITC-phalloidin. Representative images are shown. Scale bars, 50 µm. **(C)** Quantification of the data in (B). The graph shows the average length of neurite tubules. At least 50 cells were examined in each experiment. Values are expressed as means for three independent experiments ± S.D. *, $p < 0.05$, ** $p < 0.01$, (Tukey test).

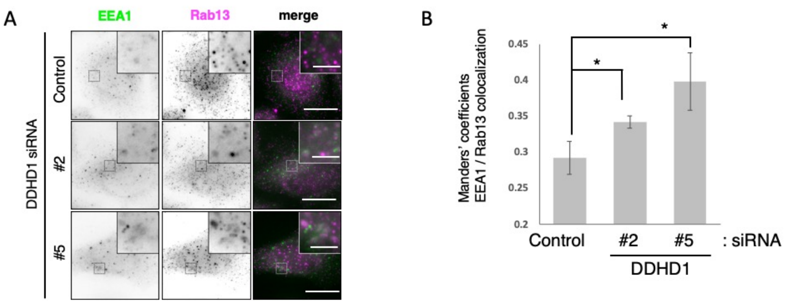

Supplementary Figure 4. DDHD1 knockdown induce co-localization between EEA1 and Rab13 in SH-SY5Y cells. (A) SH-SY5Y cells were treated with the indicated siRNAs for 72 h and subsequently stained with antibodies against EEA1 and Rab13. Higher magnification views are shown in the inset. Scale bars, 10 µm; inset, 2 µm. **(B)** Manders' colocalization analysis of the data in (A). Values are expressed as means for four independent experiments ± S.D. At least 30 cells were examined in each experiment. *, $p < 0.05$; (Tukey test).

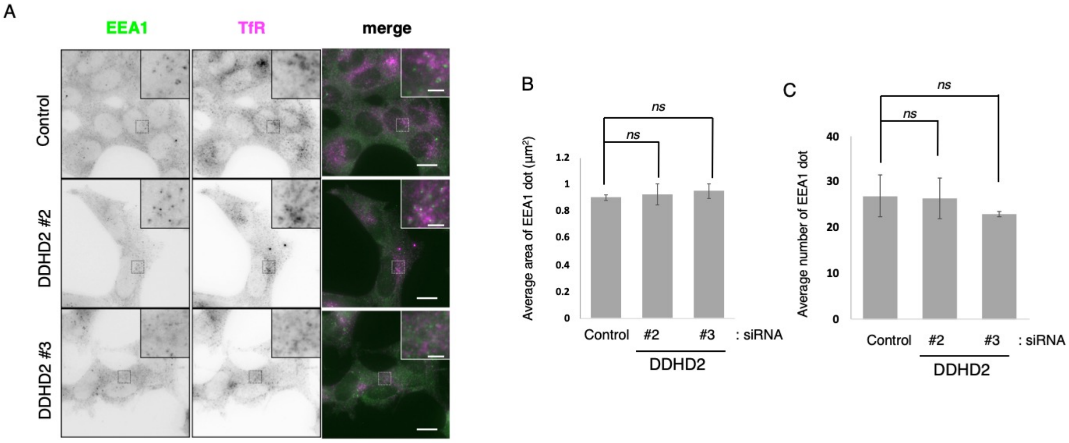

Supplementary Figure 5. Effects of DDHD2 knockdown on endosomal morphology in SH-SY5Y. (A) SH-SY5Y cells were treated with the indicated siRNAs for 72 h and subsequently stained with antibodies against EEA1 and TfR. Higher magnification views of the boxed areas are shown in the inset. Scale bars, 10 μm; inset, 2 μm. **(B,C)** The average area (B) and number (C) of early endosomes in each cell were measured under each condition. Values are expressed as means for three independent experiments ± S.D. At least 30 cells were examined in each experiment. ns; not significant (Tukey test).

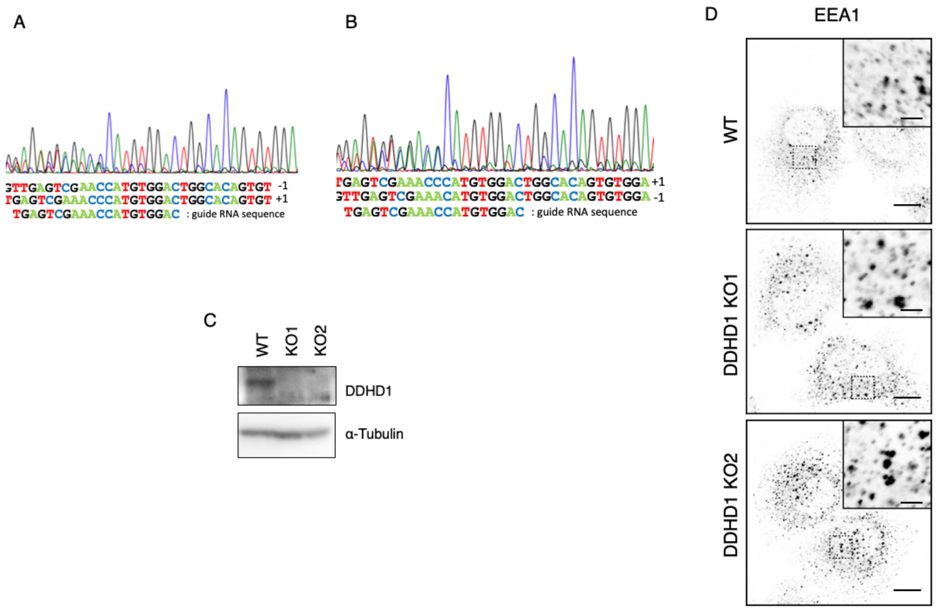

Supplementary Figure 6. Confirmation of DDHD1 KO. (A,B) Sequence results of the DDHD1 genome and guide RNA sequences are shown. **(C)** Cell lysates of HeLa or DDHD1 KO HeLa cells were subjected to Western blotting analysis with the indicated antibodies. **(D)** DDHD1 KO HeLa cells were fixed and stained with an antibody against EEA1. Higher magnification views of the boxed areas are shown in the inset. Scale bars, 10 µm; inset, 2 µm.

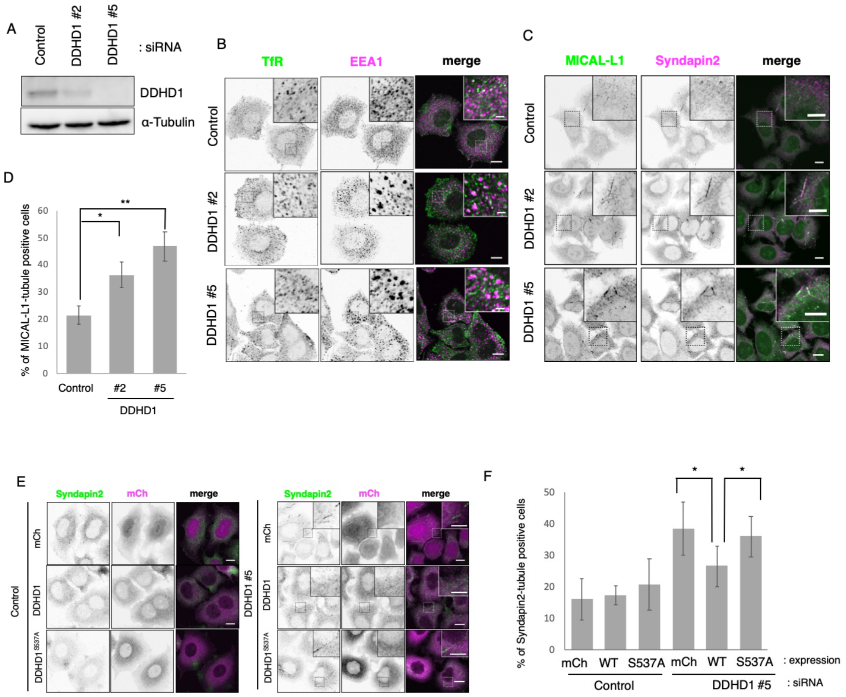

Supplementary Figure 7. Effects of DDHD1 knockdown on early endosomes and syndapin2-positive recycling tubules. (A) HeLa cells were transfected with DDHD1 siRNA #2, DDHD1 siRNA #5, or control siRNA. After 72 h, the cells were harvested and dissolved with SDS sample buffer followed by sonication and Western blotting analysis with the indicated antibodies. **(B,C)** At 72 h after siRNA treatment, the cells were fixed and stained with antibodies against TfR and EEA1 (B) or MICAL-L1 and syndapin2 (C). Higher magnification views are shown in the inset. Scale bars, 10 µm; inset, 2 µm in (B); inset, 5 µm in (C). **(D)** Quantification of the data in (C).The percentages of the cells containing MICAL-L1-positive tubules of over 3 µm are shown in the graph. **(E)** HeLa cells stably expressing mCherry, mCherry-DDHD1, or mCherry-DDHD1^{S537A} were treated with DDHD1 siRNA #5 or control siRNA for 72 h, followed by Syndapin2 staining. At least 50 cells were examined in each experiment. Higher magnification views are shown in the inset. Scale bars, 10 µm; inset, 5 µm. **(F)** Quantification of the data in (E). The percentages of the cells containing Syndapin2-positive tubules of over 3 µm are shown in the graph. Values are expressed as means for four independent experiments ± S.D. At least 30 cells were examined in each experiment. *, p <0.05; **, p <0.01; (Tukey test).

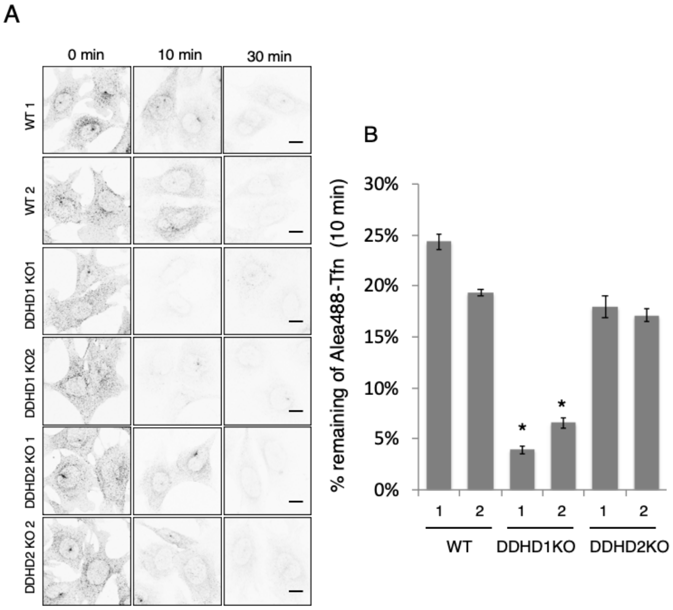

Supplementary Figure 8. Effects of DDHD1 or DDHD2 KO on recycling of Alexa-488-Tfn in MEF cells. (A) Wild-type, DDHD1 KO, or DDHD2 KO MEF cells were subjected to Alexa488-Tfn recycling assay for the indicated times as described under Experimental Procedures. Scale bars, 10 µm. **(B)** The percentages of remaining Alexa488-Tfn signals at 10 min compared to the fluorescent signals at 0 min are shown in the line graph. Values are expressed as means for three independent experiments ± S.D. *, $p < 0.05$; (Tukey test).

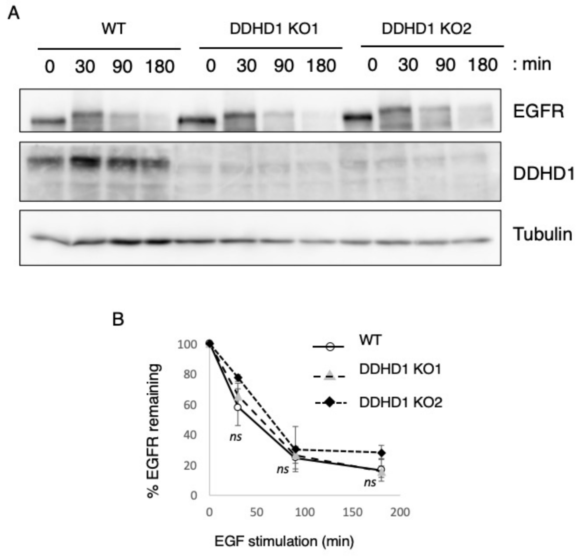

Supplementary Figure 9. DDHD1 KO does not affect the endosome-lysosome pathway. (A) Wild-type or DDHD1 KO HeLa cells were serum-starved for 3 h and then stimulated with 100 ng/ml EGF for the indicated times. The cells were harvested with SDS sample buffer, followed by sonication and Western blotting analysis with the indicated antibodies. **(B)** The intensities of the immunoreactive signals were quantified, and the percentages of EGF receptor remaining relative to that at 0 min at each time point are shown in the graph. Values are expressed as means for three independent experiments ± S.D. ns; not significant (Tukey test).
